# Intranasal Microemulsion as an Innovative and Promising Alternative to the Oral Route in Improving Stiripentol Brain Targeting

**DOI:** 10.3390/pharmaceutics15061641

**Published:** 2023-06-01

**Authors:** Sara Meirinho, Márcio Rodrigues, Adriana O. Santos, Amílcar Falcão, Gilberto Alves

**Affiliations:** 1 CICS-UBI, Health Sciences Research Centre, University of Beira Interior, Av. Infante D. Henrique, 6200-506 Covilhã, Portugal; sara.meirinho@ubi.pt (S.M.); marciorodrigues@fcsaude.ubi.pt (M.R.); asantos@fcsaude.ubi.pt (A.O.S.); 2 Faculty of Health Sciences, University of Beira Interior, Av. Infante D. Henrique, 6200-506 Covilhã, Portugal; 3 CPIRN-UDI-IPG, Center for Potential and Innovation of Natural Resources, Research Unit for Inland Development, Polytechnic of Guarda, 6300-559 Guarda, Portugal; 4 CIBIT/ICNAS, Coimbra Institute for Biomedical Imaging and Translational Research, University of Coimbra, Pólo das Ciências da Saúde, Azinhaga de Santa Comba, 3000-548 Coimbra, Portugal; acfalcao@ff.uc.pt; 5 Laboratory of Pharmacology, Faculty of Pharmacy, University of Coimbra, Pólo das Ciências da Saúde, Azinhaga de Santa Comba, 3000-548 Coimbra, Portugal

**Keywords:** albumin, brain delivery, epilepsy, intranasal administration, microemulsion, mucoadhesive, stiripentol

## Abstract

Stiripentol (STP) is a new-generation antiepileptic only available for oral administration. However, it is extremely unstable in acidic environments and undergoes gastrointestinal slow and incomplete dissolution. Thus, STP intranasal (IN) administration might overcome the high oral doses required to achieve therapeutic concentrations. An IN microemulsion and two variations were herein developed: the first contained a simpler external phase (FS6); the second one 0.25% of chitosan (FS6 + 0.25%CH); and the last 0.25% chitosan plus 1% albumin (FS6 + 0.25%CH + 1%BSA). STP pharmacokinetic profiles in mice were compared after IN (12.5 mg/kg), intravenous (12.5 mg/kg), and oral (100 mg/kg) administrations. All microemulsions homogeneously formed droplets with mean sizes ≤16 nm and pH between 5.5 and 6.2. Compared with oral route, IN FS6 resulted in a 37.4-fold and 110.6-fold increase of STP plasmatic and brain maximum concentrations, respectively. Eight hours after FS6 + 0.25%CH + 1%BSA administration, a second STP brain concentration peak was observed with STP targeting efficiency being 116.9% and direct-transport percentage 14.5%, suggesting that albumin may potentiate a direct STP brain transport. The relative systemic bioavailability was 947% (FS6), 893% (FS6 + 0.25%CH), and 1054% (FS6 + 0.25%CH + 1%BSA). Overall, STP IN administration using the developed microemulsions and significantly lower doses than those orally administrated might be a promising alternative to be clinically tested.

## 1. Introduction

Epilepsy is a chronic noncommunicable disease diagnosed in approximately 50 million people of all ages, making it the fourth more prevalent neurological disease worldwide [1]. Focal and generalized seizures are usually the most common clinical features of epilepsy conditions. However, closer attention must also be paid to epileptic syndromes. These usually start at early ages and are manifested through a variety of symptoms, later contributing to the occurrence of intellectual, psychiatric and other comorbidities [2]. One of those conditions is Dravet syndrome, a severe developmental and epileptic encephalopathy associated with detrimental variations in the sodium channel α-1 subunit gene [3,4]. Its onset is usually before 1 year old, with children frequently presenting prolonged hemiclonic seizures associated with fever [5]. Throughout patient’s life, the syndrome severity tends to increase mainly due to the high prevalence of pharmacoresistance, significantly impairing patients’ quality of life [3,4,5]. Hence, the treatment of Dravet syndrome is primarily focused on reducing the number and duration of generalized tonic–clonic seizures and preventing *status epilepticus*, thereby decreasing the risk of sudden death in children [3,6]. In this context, stiripentol (STP), a third-generation antiepileptic drug, emerged as a promising novel therapy. The European Medicines Agency fully approved STP as an orphan drug in 2014, being currently indicated as an adjunctive treatment for Dravet syndrome in children [3,6]. However, the efficacy of STP has also been clinically demonstrated in the management of focal and generalized drug-resistant epilepsies [7] and in the treatment of super-refractory *status epilepticus* [8], which might broaden its current therapeutic indication. Additionally, STP has shown promising results in diseases outside the context of epilepsy, namely in the treatment of hyperoxaluria [9], thrombocytopenia [10], and cerebral ischemia [11]. 

Chemically, STP is characterized as being practically insoluble in water (49.2 mg/L) and extremely unstable in acidic environments (e.g., gastric acid in an empty stomach) [12,13,14]. In addition to gastric instability, STP also undergoes a slow and incomplete dissolution in the gastrointestinal tract, an extensive first-pass hepatic metabolism mediated by different cytochrome P450 isoenzymes and presents a high potential for drug interactions. Thus, oral STP absorption and overall bioavailability are expected to be highly limited [12,15]. Nevertheless, STP is currently only available as oral capsules and powder for oral suspension [16]. Hence, in order to achieve plasmatic therapeutic concentrations (4–22 mg/L), high doses of STP need to be administered [13,17]. This justifies the prescription of scaling doses between 20 mg/kg/day and 50 mg/kg/day up to a maximum of 4 g per day administered in 12/12 h or 8/8 h dosing schedules [16]. 

Bearing in mind the therapeutic potentialities of STP, alternatives that could increase STP bioavailability need to be pursued. In fact, several authors have developed alternative formulations for the administration of STP [12,15,18,19]; however, even considering all its limitations [20], the oral route continued to be the only route investigated for STP administration so far. In this context, the intranasal (IN) delivery of STP arises as a hopeful alternative to be explored in order to overcome the several aforementioned disadvantages associated with its oral administration. In fact, the anatomophysiological characteristics of the nasal cavity partially permit a direct nose-to-brain transport of drugs through the trigeminal and olfactory nerve, circumventing the blood–brain barrier (BBB) [21,22,23,24]. In addition to it, intranasally administered drugs can also reach the brain after being systemically absorbed into the bloodstream and cross the BBB, circumventing the first-pass metabolism in the gut and liver [21,22,25]. The physiological pH of the nasal cavity (5.0–6.5) also makes the IN route attractive for the administration of drugs that are unstable at extreme pH values, as is the case of STP [20,21]. Taking all this into account, we hypothesized that an increase in STP brain bioavailability could occur after its IN administration using lower doses than those orally administered. 

Although the IN route presents a high number of advantages, an intrinsic disadvantage is the small volume of the nasal cavity. Thus, since STP is not a very potent antiepileptic drug, it is essential to develop high drug-strength formulations that enable the administration of the intended dose in a small volume of formulation [21,26,27]. In this context, lipidic nanosystems are presented as an attractive approach for STP formulation, since they are commonly investigated to improve properties that lead to efficient nose-to-brain delivery. In fact, nanometric emulsions as micro- and nanoemulsions can maintain a high content of lipophilic molecules in a solubilized state, with a simultaneous increasing its stability [20,28]. In terms of pharmaceutical characteristics, if droplets are homogeneously distributed [polydispersity index (PDI) < 0.1] and present a mean size of ≤100 nm, the transport of the loaded drugs from the nasal cavity to the brain can be facilitated, preventing its distribution to nontarget organs [20,29]. Viscosity should also be adequate to enhance the residence time of formulations without compromising cilia’s regular function [20,22,23]. For that, an adequate approach can be the incorporation of generally regarded as safe (GRAS) mucoadhesive polymers such as chitosan or carbopol. [24,30,31]. In addition to the mucoadhesive property, chitosan also causes a reversible opening of the epithelium tight junctions, potentially enhancing drug permeability and increasing its extracellular transport to the brain [31]. For those reasons, several antiepileptics were already formulated in mucoadhesive emulsions either using carbopol [32,33,34] or chitosan [35]. It is also reported that albumin can be transported by active mechanisms from the nasal cavity to the brain. Thus, the addition of a percentage of albumin to the formulations’ composition can be another interesting approach to increase the brain bioavailability of drugs administered by the IN route [36,37,38]. This is mostly valid for nose-to-brain delivery of drugs characterized for their high affinity to plasmatic proteins, as it is the case of STP [16].

In this study, the aim was to develop a nanometric emulsion capable of delivering STP from the nose cavity to the brain using a smaller dose than that administered by oral route. With this approach, it is expected to overcome the problems associated with STP acid instability together with the slow and incomplete dissolution in the gastrointestinal tract and its first-pass hepatic effect. Consequently, an enhancement in brain bioavailability and therapeutic effect is expected by using a more cost-effective and patient-friendly alternative to administer STP.

## 2. Materials and Methods

### 2.1. Materials

STP (≥98% purity) was purchased from Tokyo Chemical Industry (Tokyo, Japan). Terbinafine hydrochloride (99.5% purity), used as an internal standard in the chromatographic analysis, was kindly supplied by Bluepharma (Coimbra, Portugal). Pentobarbital sodium injection solution (Eutasil^®^) was purchased from Ceva (Libourne, France). Imwitor^®^ 988 was donated by IOI Oleochemical (Hamburg, Germany); Transcutol^®^ HP and Capryol^®^ 90 were kindly supplied by Gattefossé (Saint-Piest, France); Kolliphor^®^ RH 40, Kolliphor^®^ EL, Tween 80 and Kollisolv^®^ PG were gift samples from BASF Europe. Capmul^®^ MCM and Acconon CC-6 were obtained from ABITEC (Columbus, OH, USA); chitosan (batch no. 141276-O-2), polyethylene glycol 400 (PEG 400) and Tween 20 from Acofarma (Barcelona, Spain); the carbomer Carbopol^®^ 971P from Lubrizol (Brussels, Belgium) and dimethyl sulfoxide (DMSO), carboxymethylcellulose sodium salt, and bovine serum albumin (BSA) were obtained from Sigma-Aldrich (St. Louis, MO, USA). Acetonitrile (HPLC grade), methanol (HPLC grade), analytical grade triethylamine, and 85% *ortho*-phosphoric acid were all purchased from Fisher Scientific (Leicestershire, UK). Isopropanol (98% purity) and absolute ethanol (99.9% purity) were supplied from Honeywell Riedel-de Haën™ (Seelze, Germany); magnesium chloride, sodium chloride, sodium carbonate, potassium chloride, and acetic acid glacial (99–100%) were purchased from Chem-Lab (Zedelgem, Belgium); sodium phosphate dibasic anhydrous (98% purity) and sodium phosphate monobasic anhydrous (98–100.5% purity) were both supplied by Acros Organics (Morris Plains, NJ, USA); Panreac (Barcelona, Spain) was the supplier of calcium chloride and magnesium sulphate (MgSO_4_); Fluka (Seelze, Germany) was the provider of hydrochloric acid fuming 37%, and sodium chloride solution (NaCl) 0.9% acquired from B. Braun Medical (Queluz de Baixo, Portugal). A Milli-Q water apparatus from Millipore (Milford, MA, USA) was used to prepare the ultrapure water (HPLC grade, >18 MΩ.cm).

### 2.2. Solubility Studies

To formulate STP in a nanometric emulsion that could include a mucoadhesive agent, different mixtures of excipients (oils, surfactants, and cosurfactants) were tested, with final percentages of aqueous phase between 10% and 85% (Table 1). This study was carried out based on the composition of nanometric emulsions previously issued in the literature for the IN administration of lipophilic drugs, modifying some excipients and their corresponding proportion [32,35,39,40,41,42]. Emulsions were independently prepared by weighing together the oil, surfactant, and cosurfactant (anhydrous phase) of each formula. Then, the corresponding water percentage (external phase) was added dropwise, with final gentle mixing. To address STP solubility, an excess amount of STP powder (300 mg) was added to 1 mL of each prepared formulation. Then, the samples were mixed using a vortex and shaken for 48 h at 300 rpm in an orbital shaker incubator thermostatized at 25 ± 1 °C. Finally, the mixtures were centrifuged at 12,300 g for 20 min and supernatants were recovered to quantify the STP concentrations. Each supernatant sample (*n* = 3) was first diluted 100-fold with methanol followed by a second 100-fold dilution in a mixture composed of 53% (*v*/*v*) water plus 47% (*v*/*v*) acetonitrile, and only then analysed by an HPLC method [43]. 

### 2.3. Preparation of the Most Promising Nanometric Emulsions

Considering the solubility results (Section 2.2), the formulation that better solubilized STP (FS6) was prepared for further characterization and mucoadhesive incorporation. The emulsions, containing or not 75 mg/mL of STP, were prepared by the water titration method. FS6 was prepared by weighting together Capmul MCM (oil), Kolliphor RH40 (surfactant) and Transcutol HP (cosurfactant), completing with a corresponding percentage of distilled water (Table 1). To incorporate the drug, STP was solubilized in the anhydrous preconcentrate. Then, the aqueous phase was added dropwise under continuous gentle stirring. Mucoadhesive emulsions were prepared by adding an aqueous dispersion of Carbopol 971P or a 1% (*v*/*v*) acetic acid solution of chitosan to the anhydrous preconcentrates in order to achieve a final concentration of 0.5% (*w*/*w*) or 0.25% (*w*/*w*). Later, the addition of BSA to FS6 containing 0.25% (*w*/*w*) chitosan was accomplished by dissolving the appropriate BSA mass in the aqueous phase of the respective formulation to reach the final BSA concentration of 1% (*w*/*v*).

### 2.4. Pharmaceutic Characterisation of the Selected Formulations

#### 2.4.1. Mean Droplet Size and Polydispersity Index

The mean hydrodynamic droplet diameter (droplet size) and PDI of each preparation, with or without mucoadhesive agents, were measured after a 100-fold dilution in ultra-pure water. Samples were automatically measured in triplicate at 25 °C using disposable ultraviolet/visible polymethyl methacrylate cuvettes (Kartell, Noviglio, Italy) and a Zetasizer Nano ZS apparatus (Malvern, UK). Mean droplet size and PDI were automatically obtained using an analysis software that calculated these parameters by cumulants’ analysis of dynamic light-scattering data.

#### 2.4.2. Osmolarity, pH, and Rheology

Osmolality was determined using a freezing-point osmometer (Osmomat 3000, Gonotec, Berlin, Germany) and mean values of three measurements (*n* = 3) were calculated for each formulation.

The pH was measured using a calibrated pH meter (Orion Star A211 pH meter, Thermo Fisher Scientific, New Hampton, NH, USA).

Viscosity was assessed by measuring 0.5 mL of each emulsion in a Brookfield DV3T cone-plate rheometer coupled with either a CP40Z or a CP52Z cone spindle (Brookfield Ametek, Massachusetts, United States of America). The temperature was regulated and kept constant at 20 °C (room temperature) or at 32 °C (nasal-cavity temperature) by a water bath (MultiTemp III Thermostatic Circulator, Thermo Fisher Scientific, New Hampton, NH, USA). For Newtonian fluids, zero shear viscosity was considered to be the continuous viscosity value reached when a maximum torque value (less than 100%) at a respective shear rate was attained. For fluids presenting a non-Newtonian pseudoplastic behaviour, zero-shear viscosity was statistically estimated using the measurements obtained at different shear rates at a temperature of 20 °C or 32 °C.

#### 2.4.3. Assessment of STP In Vitro Release

STP in vitro release was assessed in horizontal Ussing chambers (Harvard Apparatus, NaviCyte, Hugstetten, Germany) using a protocol previously developed by our research group [44], with some modifications that ensured the experimental sink conditions for STP. The concentration of STP in each tested formulation that was added to the donor chamber (100 μL) at the beginning of each assay was of 5 mg/mL. At predetermined time points after starting the test (10, 30, 60, 90, 120, 150, 180, 240, and 300 min) samples of each receptor chamber (200 μL) were removed for STP chromatographic quantification. For that, each collected sample was 25-fold diluted using mobile phase [water/acetonitrile (53:47, *v*/*v*)] and then injected into the HPLC apparatus. Each formulation added to the donor compartments was also quantified, preceded by a first 100-fold dilution in Transcutol HP followed by a second 25-fold dilution using a mixture composed of 53% (*v*/*v*) water plus 47% (*v*/*v*) acetonitrile. A simple solution of STP in Transcutol HP (5 mg/mL) was used as a positive control of drug release. 

### 2.5. In Vivo Pharmacokinetic Studies

#### 2.5.1. Animals

Healthy adult male CD-1 mice, aged between 8 and 10 weeks and weighing from 30 to 45 g, were supplied by the Faculty of Health Sciences of the University of Beira Interior (Covilhã, Portugal), a certified animal facility. Controlled environmental conditions were always maintained during the animals’ housing (12 h light/dark cycles, 20 ± 2 °C, and 50 ± 5% relative humidity), with tap water and standard rodent diet given ad libitum (4RF21, Mucedola, Italy). The animal experimental protocols were approved by the local Animal Welfare and Ethical Review Body, in agreement with European Directive 2010/63/EU regulations transposed to Portuguese legislation (Decree-Law no. 113/2013).

#### 2.5.2. Single-Dose Pharmacokinetic Studies

For pharmacokinetic studies, male CD-1 mice were randomly divided into five independent groups: the first three groups received IN administrations of STP (5 μL/30 g body weight) incorporated in either FS6, FS6 plus 0.25% chitosan (for now on designated FS6 + 0.25%CH), or FS6 plus 0.25% chitosan plus 1%BSA (for now on designated FS6 + 0.25%CH + 1%BSA); the fourth group was orally dosed (300 μL/30 g body weight) with a 0.5% (*w*/*v*) sodium carboxymethylcellulose aqueous STP suspension using an appropriate oral gavage apparatus; and the fifth group was dosed with a slow (over approximately 1 min) intravenous (IV) tail-vein injection (60 μL/30 g of body weight) of an STP solution composed by 50% (*v*/*v*) propylene glycol, 30% (*v*/*v*) NaCl 0.9%and 20% (*v*/*v*) ethanol. The STP dose administered by IN and IV routes was 12.5 mg/kg and the oral dose was 100 mg/kg. For mice immobilization during IN and IV administrations, a pentobarbital (60 mg/kg) intraperitoneal (IP) injection was given to all the animals of the IN and IV groups, being then maintained in a warm environment to avoid hypothermia. To perform IN administrations, sedated mice were placed in a supine head-back position on top of a rolled pillow set in a heating pad coupled with a DC Temperature Controller 40-90-8D (FHC, Bowdoin, Maine, USA). A flexible polyurethane catheter coupled to a 50 μL syringe (Hamilton, NV, USA) was inserted 3 to 4 mm into the right nostril. After the IN STP drug, sedated mice were left to recover in a temperature-controlled environment to avoid hypothermia. 

After 5, 10, 15, 30, 45, 60, 90, 120, 180, 240, 360, 480, 600, and 720 min of STP administrations, (*n* = 4 per time point), cardiac puncture was performed on anesthetised mice in order to collect around 1 mL of blood to K_3_EDTA tubes (BD Vacutainer^®^). Immediately after blood collection, mice were decapitated, and each brain was harvested and gently washed using a 0.9% NaCl solution to remove all the signs of blood. Blood samples were centrifuged at 3351× *g* for 10 min at 4 °C to obtain plasma samples. Plasma and brain samples were stored at −20 °C protected from light until analysis.

#### 2.5.3. Processing of Biological Samples and HPLC Analysis

After thawing, brains were weighed and homogenized in a tissue homogenizer (Ika Ultra-Turrax^®^ T25 Basic, Staufen, Germany) after the addition of 4 mL of 1 M sodium phosphate buffer pH 5 per gram of tissue. Brain homogenates were centrifuged at 17,350× *g* for 10 min at 4 °C and the corresponding supernatants were collected for analysis. 

All quantifications of STP in the plasma and brain samples obtained from the in vivo pharmacokinetic studies were performed by means of a fully validated HPLC method previously developed by our research group [43]. As reported in the Meirinho et al. [43] study, each sample of plasma and brain (100 μL) was prepared by a validated salting-out liquid–liquid extraction procedure. Then, each obtained extracted sample was analysed by a validated reversed-phase HLPC quantification method coupled with a fluorescence detector. 

#### 2.5.4. Pharmacokinetics Analysis and Calculation

STP maximum concentration (C_max_) in the plasma and the brain, and the correspondent time to attain it (t_max_), were directly estimated from the experimental data. All remaining pharmacokinetic parameters were calculated using WinNonlin version 5.2. (Pharsight Co., Mountain View, CA, USA). To reduce the bias of the estimated parameters, a noncompartmental study approach was applied only considering the mean concentrations (*n* = 4) at each time postdosing, without assuming any exponential function. Plasma and brain concentrations below the lower limit of quantification of the analytical method (10 ng/mL) [43] were considered as zero in the pharmacokinetic data analysis. The area under the curve of the plasma and brain concentration versus time profile (AUC) from time zero to the time of the last quantifiable sampling (AUC_0-t_) was calculated based on the linear trapezoidal rule. The AUC extrapolation to infinity (AUC_inf_) was calculated using the formula AUC_0-t_ + (C_last_/k_el_), where C_last_ corresponds to the last quantifiable sample concentration and k_el_ is the terminal elimination rate constant calculated using a log-linear regression of the terminal portions of each plasma and brain concentration versus time profile. The time needed for STP plasmatic and brain concentrations to decrease by one half [elimination half life (t_1/2el_)] was calculated from the k_el_ value through the formula ln2/k_el_. The mean time that STP stayed in the body [mean residence time (MRT)] was calculated as the ratio between the area under the first moment curve and the corresponding AUC_0-t_. The extrapolated percentage of the AUC [AUC_extrap_ (%)] from the time of C_last_ to infinity, was calculated as [(AUC_inf_ − AUC_0-t_)/AUC_inf_] × 100. To secure the collection of samples during sufficient time in order to properly describe the STP pharmacokinetics, AUC_extrap_ should be preferably less than 20%.

Plasmatic absolute bioavailability (F) and relative bioavailability (F_rel_) of STP after IN administration of the tested nanometric emulsions and after oral suspension administration were calculated according to Equations (1) and (2), respectively:

(1) $$F=\frac{{\mathrm{AUC}}_{\inf-\mathrm{IN}\;\mathrm{or}\;\mathrm{Oral}}\times {\mathrm{Dose}}_{\mathrm{IV}}}{{\mathrm{AUC}}_{\inf-\mathrm{IV}}\times {\mathrm{Dose}}_{\mathrm{IN}\;\mathrm{or}\;\mathrm{Oral}}}\times 100$$

(2) $$F_{\mathrm{rel}}=\frac{{\mathrm{AUC}}_{\inf-\mathrm{IN}}\times {\mathrm{Dose}}_{\mathrm{Oral}}}{{\mathrm{AUC}}_{\inf-\mathrm{Oral}}\times {\mathrm{Dose}}_{\mathrm{IN}}}\times 100$$

To evaluate STP brain accumulation and compare it with plasma after its IN, IV, and oral administrations, the AUC_0-t brain_/AUC_0-t plasma_ ratios were calculated and follow compared.

To understand the general tendency of STP to be delivered to the brain after IN administration of FS6, FS6 + 0.25%CH, and FS6 + 0.25%CH + 1%BSA vs. IV administration, the drug targeting efficiency (%DTE) percentage was calculated following Equation (3):

(3) $$(\%\mathrm{DTE})=\frac{{\left(\frac{{\mathrm{AUC}}_{0-t\;\mathrm{brain}}}{{\mathrm{AUC}}_{0-t\;\mathrm{plasma}}}\right)}_{\mathrm{IN}}}{{\left(\frac{{\mathrm{AUC}}_{0-t\;\mathrm{brain}}}{{\mathrm{AUC}}_{0-t\;\mathrm{plasma}}}\right)}_{\mathrm{IV}}}\times 100$$

The (%DTE) values can range from -∞ to +∞. If DTE presents a value higher than 100%, that indicates a more efficient brain targeting through IN administration than by IV injection. On the contrary, values below 100% indicate the opposite [45,46]. 

Nose-to-brain direct-transport percentage (%DTP) of STP was also calculated. It estimates the drug quantity that directly reaches the brain through trigeminal and/or olfactory nerves compared with its total delivery to the brain by direct and indirect pathways (Equation (4)):

(4) $$\mathrm{DTP}\;\left(\%\right)=\frac{{\mathrm{AUC}}_{\mathrm{brain}-\mathrm{IN}}-\left(\frac{{\mathrm{AUC}}_{\mathrm{brain}-\mathrm{IV}}}{{\mathrm{AUC}}_{\mathrm{plasma}-\mathrm{IV}}}\times {\mathrm{AUC}}_{\mathrm{plasma}-\mathrm{IN}}\right)}{{\mathrm{AUC}}_{\mathrm{brain}-\mathrm{IN}}}\times 100$$

DTP (%) values can theoretically range from −∞ to 100%. If DTP values are under zero, that points to a more efficient drug delivery to the brain following systemic routes rather than IN direct routes. Contrary, if DTP calculation results in values higher than zero, that might indicate an occurrence of STP direct delivery from the nasal cavity to the brain through trigeminal and/or olfactory routes [45,46].

The bioavailability of STP in the brain was compared after IN, IV, and oral administrations (%B_brain IN/IV_ and %B_brain IN/oral_, respectively), being the respective values calculated using brain AUC_0-t_ dose-normalized parameters. This allows for an understanding of the STP accumulation in the brain after IN delivery over the IV and oral routes, without considering plasma AUC_0-t_ values (Equations (5) and (6)):

(5) $${\%B}_{\mathrm{brain}\;\mathrm{IN}/\mathrm{IV}\;}=\frac{{\mathrm{AUC}}_{\mathrm{brain}\;\mathrm{IN}}}{{\mathrm{AUC}}_{\mathrm{brain}\;\mathrm{IV}}}\times 100$$

(6) $${\%B}_{\mathrm{brain}\;\mathrm{IN}/\mathrm{oral}\;}=\frac{{\mathrm{AUC}}_{\mathrm{brain}\;\mathrm{IN}}}{{\mathrm{AUC}}_{\mathrm{brain}\;\mathrm{oral}}}\times 100$$

Values higher than 100 indicate a stronger accumulation of STP in the brain after IN administration than after IV and oral dosages.

### 2.6. Statistical Analysis

GraphPad Prism software, version 8.0, was used for statistical data analysis and graphical representations. Differences were considered statistically significant when the *p*-values were less than 0.05 (* *p* ˂ 0.05, ** *p* ˂ 0.01, *** *p* ˂ 0.001, and **** *p* ˂ 0.0001).

A two-way ANOVA, followed by a Dunnett’s multiple comparisons post hoc test, was applied to compare mean droplet size, PDI, and STP concentration values 30 and 90 days after the preparation of the formulations (stability test).

Zero shear viscosity of non-Newtonian pseudoplastic fluids was estimated by fitting a nonlinear regression model (one phase decay) to the “viscosity vs. shear rate” data. Then, the function zero (Y when X = 0) was determined and considered as the value of zero shear viscosity of the respective formulation. 

To analyse the in vitro drug-release assay, parameters were determined considering the STP concentration in each tested formulation assayed. Drug release rates were determined using a zero-order kinetic model represented as Y=kX+b [47,48], with X being time (h), Y STP release percentage, and k the zero-order release constant that, in this case, is the rate at which STP is released from each formulation. Y values were normalized as Y = Y/0.64, considering that the membrane area is 0.64 cm^2^. A linear regression was then applied using mean normalized Y values obtained at each time point. In the case of STP release from the positive control solution, the values obtained at later time points were excluded since they fell out of the linear zone. In this way, the positive control results were also fitted into a zero-order model and then better compared with the remaining results. To assess if the calculated rates were significantly different between each formulation and between the positive control and the formulations, the drug release rates were compared two by two using an F-test.

In vivo pharmacokinetic data was expressed as mean ± standard error of the mean (SEM). A two-way ANOVA analysis with Tukey’s multiple comparisons post hoc test was used to study possible statistical differences among the three IN nanometric emulsions and between IN formulations, IV, and dose-normalized oral route at each time point after the administrations (Supplementary Table S1). 

## 3. Results

### 3.1. Characterization of the Selected Nanometric Emulsions

In order to maximize STP strength in a liquid lipidic nanosystem, STP solubility was tested in different nanometric emulsions containing aqueous percentages ranging from 10% to 85%. The composition of the prepared nanometric emulsions and the respective experimental STP solubility are described in Table 1. FS6 was the formulation that better solubilized STP, being, therefore, selected for extensive pharmaceutical characterization. After mixing the anhydrous preconcentrate with the aqueous external phase, a transparent emulsion with no visible particles or aggregates was spontaneously formed, features that are particular of a microemulsion system [20]. 

With the purpose of conferring mucoadhesiveness to FS6, different percentages of chitosan or carbopol were added to the aqueous external phase. Table 2 shows the results of FS6 pharmaceutical characterization, with and without 0.5% (*w*/*w*) or 0.25% (*w*/*w*) of the mucoadhesive polymers chitosan or carbopol. In a more advanced stage, the influence of adding 1% (*w*/*v*) albumin to FS6 containing 0.25% chitosan was also evaluated.

FS6 formed highly homogeneously distributed droplets (PDI of 0.066 ± 0.009) with a small mean size of 13.21 ± 0.09 nm, a value that fits in the microemulsion mean droplet sizes range (10–100 nm) [22,26]. After adding 0.25% of chitosan to FS6 composition, the mean droplet size only had a slight increase from 13.21 ± 0.09 nm to 14.15 ± 0.12 nm, maintaining the characteristics of a microemulsion. The PDI value was also kept in preferential values of around 0.1. However, when 0.25% of carbopol was added to FS6, its mean size and PDI increased 8.1-fold and 3.7-fold, respectively (Table 2). Regarding pH, all formulations present values inside the pH range of the nasal cavity (5.0–6.5). Since chitosan is only soluble in acidic solutions (e.g., 1% acetic acid), its addition to FS6 caused a slight decrease in the pH values. However, the obtained values continued to be within the acceptable pH range of the nasal cavity (Table 2), reducing the probability of nasal mucosa irritation and ensuring some safety degree of the microemulsion-containing chitosan. As for osmolality, either FS6 and FS6 containing different percentages of chitosan and carbopol revealed to be hyperosmotic after a twofold dilution in NaCl 0.9%, with most of the prepared formulations having osmolality values outside the safety range of 300 to 700 mOsmol/kg [49]. However, it must be considered that Transcutol HP is an excipient that has a higher impact in rising osmolarity after being diffused to the aqueous external phase, even not being expected to increase to the in vivo tonicity. Furthermore, the osmolality of the NaCl 0.9% solution used to dilute formulations must also be considered, thus inflating the final osmolality values. 

**Figure 1:**
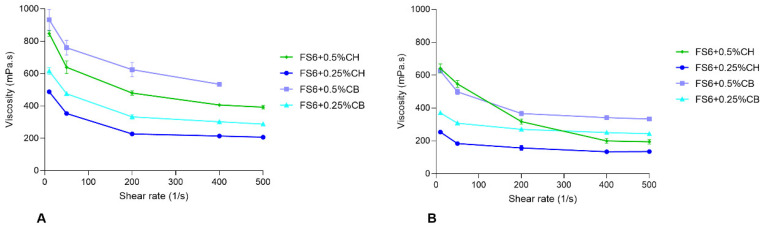
Assessment of formulations’ viscosity versus the shear rate at 20 °C (**A**) and 32 °C (**B**) of FS6 containing different percentages (0.5% or 0.25%) of the mucoadhesive polymers carbopol (CB) or chitosan (CH). Data are represented as mean ± standard error of the mean (SEM).

The rheological behaviour of FS6 was also evaluated at 20 °C (average room temperature) and 32 °C (average temperature of the nasal cavity). FS6 presents characteristics of a Newtonian fluid, with constant viscosity over a range of shear rates (124.1 ± 0.47 mPa·s, Table 2). However, with the addition of chitosan or carbopol, FS6 acquired a non-Newtonian shear-thinning behaviour, at both tested temperatures (Figure 1).

A nonlinear regression model was used to calculate the zero-shear viscosity of each formulation in study (Table 2). By analysing the obtained values, it becomes evident that, for FS6, the addition of 0.5% chitosan or carbopol was translated in higher zero-shear viscosities. This is even more evident at 20 °C than at 32 °C. Therefore, since the more viscous formulations containing 0.5% of mucoadhesive polymers present zero-shear viscosity values outside the theoretical optimal values for nasal formulations (500 mPa∙s) [50], it is possible that those formulations might increase nasal residence time but decrease STP diffusion during nose-to-brain delivery. 

In a next phase, the addition of 1% (*w*/*v*) albumin to FS6 plus 0.25% chitosan was evaluated. A slight increase in visual formulation turbidity was observed, accompanied by an increase in the PDI value. However, no changes in mean droplet size, osmolality, and pH values were observed when compared with the respective formulation without 1% albumin (Table 2). 

**Figure 2:**
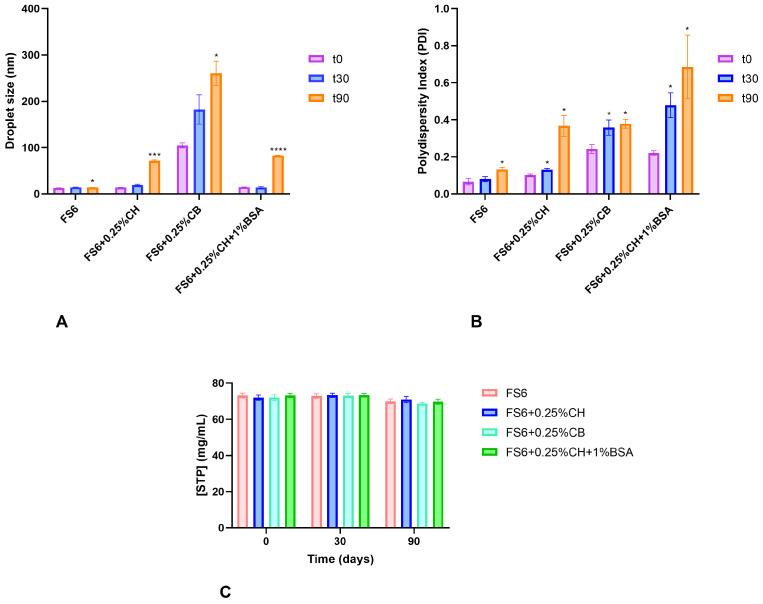
Mean hydrodynamic droplet size (**A**), PDI (**B**) and stiripentol (STP) concentration (**C**) values variation between 0 (t0), 30 (t30), and 90 (t90) days after the preparation of FS6 containing 0.25% of chitosan (CH) or carbopol (CB). The addition of 1% albumin to FS6 containing 0.25% chitosan (FS6 + 0.25%CH + 1%BSA) was also the target of stability evaluation. Values expressing mean ± standard error of the mean (SEM) of three measurements per time point of each formulation batch. PDI, polydispersity index. * *p* < 0.05, *** *p* < 0.001, **** *p* < 0.0001.

Physical stability of FS6, with and without mucoadhesive polymers, was also evaluated at room temperature. Mean droplet size and PDI values were obtained right after preparation and after 30 and 90 days of its preparation (Figure 2A,B). For all cases, after 90 days, a statistically significant increase in mean droplet size and PDI was observed, indicating that better long-term storage conditions might be required. The chemical stability of STP in each microemulsion was also evaluated under the same conditions. No significant changes in STP concentrations were observed in each tested microemulsion over the 90 days of evaluation (Figure 2C).

### 3.2. Assessment of STP In Vitro Release

**Figure 3:**
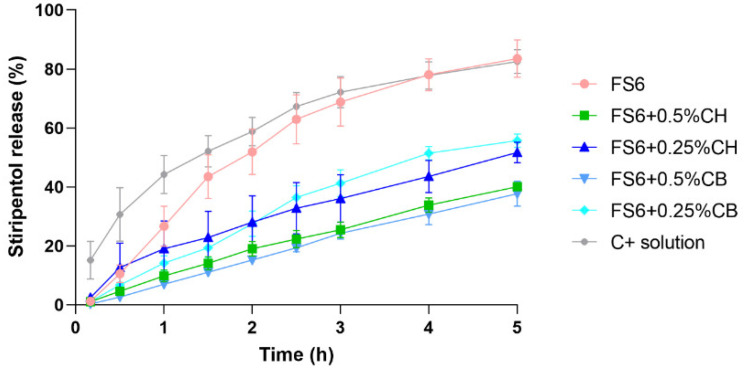
Percentual drug release of stiripentol (STP) over the 5 h of in vitro testing from FS6 and FS6 containing different percentages (0.5% or 0.25%) of the mucoadhesive polymers carbopol (CB) or chitosan (CH). Symbols represent the mean ± SEM values obtained in the four chambers used (*n* = 4). C+ solution was prepared in Transcutol HP and used as a positive control.

To further support the formulation selection for the in vivo pharmacokinetic assays, the in vitro drug release profiles were evaluated using horizontal Ussing chambers. FS6 (5 mg/mL of STP), containing or not 0.25% or 0.5% chitosan or carbopol, were tested. An STP solution in Transcutol HP (5 mg/mL) was used as a positive control of drug release. The cumulative percentages of the release profiles of STP and the STP release rates are shown in Figure 3 and Table 3, respectively. Several kinetic models were fitted to the obtained data, with the zero-order kinetic model being the one that best fitted to all formulations. Thus, STP release rates were calculated by fitting the normalized results (Y = Y/0.64) to a zero-order kinetic model that was further analysed by linear regressions (within the linear range), with differences being compared by applying an F-test.

By analysing Figure 3, it is clear that the STP release from the Transcutol HP solution was not complete, reaching a plateau of around 80%. That could mean that the remaining 20% might be retained either through adsorption or in the donor chamber/membrane. Still, when looking at the calculated release rates in Table 3, it is notorious that STP was released faster from the Transcutol HP solution than from the tested nanometric emulsions. Only FS6 showed a nonsignificant difference when compared with the STP solution, resulting in similar release rate values of, respectively, 27.15 ± 3.23%∙cm^−2^∙h and 32.71 ± 3.73%∙cm^−2^∙h. Considering the mucoadhesive formulations, a decrease in both the percentage of STP release (Figure 3) and in the STP release rate (Table 3) were obtained. That was even more evident when the final percentages of chitosan and carbopol increased from 0.25% to 0.5%. A plausible justification might be the higher zero-shear viscosity values of the formulations containing higher percentages of mucoadhesive polymers. In fact, a higher viscosity can be responsible for decreasing drug diffusion and release rate, even if it allows a longer retention time in the nasal cavity. Therefore, a balanced effect between formulation viscosity and release rate needs to be accomplished to increase drug bioavailability after nasal administration.

Considering the pharmaceutical characterization of the studied formulations (mean droplet size, PDI, pH, osmolarity, viscosity, and in vitro release), the formulations FS6 and FS6 + 0.25%CH that demonstrated characteristics of microemulsions were herein chosen to be tested in vivo. The main reasons for this choice rely on the small droplet size and the corresponding homogenous distribution of FS6 and FS6 + 0.25%CH; in the pH values that demonstrated to be within the acceptable range for the nasal cavity; in enough viscosity of both formulations that potentially lead to a mucoadhesive effect, but still allow an acceptable STP percentage and rate release; and in the greater stability after 30 and 90 days compared to the formulations containing carbopol. Furthermore, and in order to understand the in vivo influence of including albumin in a mucoadhesive formulation, BSA was added to FS6 + 0.25%CH in a final percentage of 1% (*w*/*v*). This formulation also showed good pharmaceutical characteristics that included a mean droplet size in the microemulsion range. All selected formulations allowed the solubilization of 75 mg/mL of STP, a suitable concentration for the nasal dosing of 12.5 mg/kg in a volume of 5 μL/30 g mice weight.

### 3.3. Stiripentol Pharmacokinetic Results

**Figure 4:**
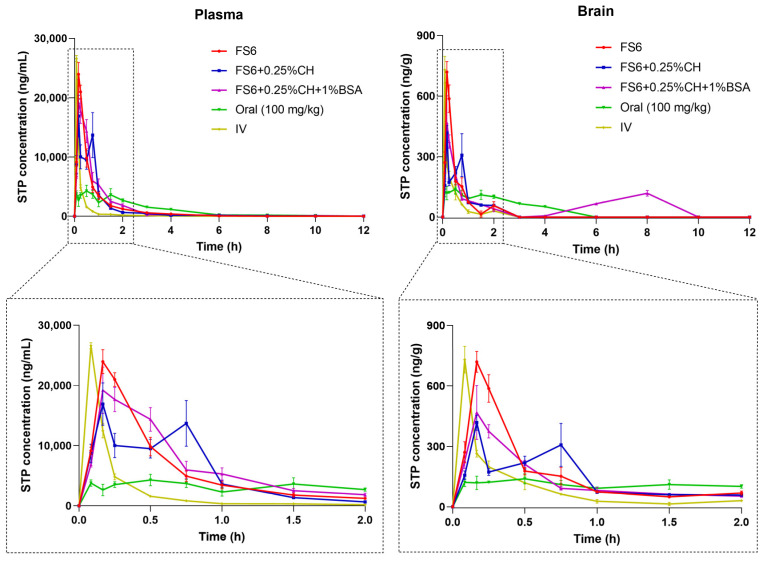
Concentration-time profiles of stiripentol (STP) in plasma and brain until 12-h postdosing after intravenous (IV, 12.5 mg/kg), oral (100 mg/kg), and intranasal [IN, FS6, FS6 plus 0.25% chitosan (FS6 + 0.25%CH) and FS6 plus 0.25% chitosan and 1% albumin (FS6 + 0.25%CH + 1%BSA) at 12.5 mg/kg] administrations to different mice groups. The figure below for each complete profile represents the amplification until 120 min post-dosing. Symbols represent the mean values ± SEM of the mice tested at each time point (*n* = 4).

The concentration-time profiles of STP in mice plasma and brain after IV (12.5 mg/kg), oral (100 mg/kg), and IN administration using the microemulsions FS6, FS6 + 0.25%CH, and FS6 + 0.25%CH + 1%BSA (12.5 mg/kg) were obtained and compared (Figure 4 and Supplementary Table S1). Table 4 shows the respective calculated pharmacokinetic parameters. Since the oral dose was eightfold higher than the dose administered by the IV and IN routes, dose-normalized pharmacokinetic parameters were also calculated (Table 4).

As expected, the STP C_max_ in the plasma and brain was reached faster after IV administration comparatively with IN and oral dosing (Figure 4). Nevertheless, after IN administration of all tested microemulsions, the plasmatic and brain t_max_ of STP was reached 10 min post-dose, a much faster time than the 30 min obtained after oral administration. 

Similar values of STP C_max_ in the plasma and brain were achieved with IV and IN administration of FS6 but not with the administration of the two nasal mucoadhesive formulations. However, the plasmatic and brain AUC_0-t_ and AUC_inf_ values after IV dosing were much lower than the obtained after IN administration using the three tested microemulsions. In fact, the IN absolute bioavailability (F) was 271.0% for FS6, 255.5% for FS6 + 0.25%CH, and 301.6% for FS6 + 0.25%C + 1%BSA (Table 5), demonstrating that, after IN administration, there is a higher amount of STP available in blood circulation to achieve the brain through the BBB than after IV administration. In concordance, all calculated %B_brain IN/IV_ values were higher than 100% (Table 5), demonstrating a higher STP accumulation in the brain (biophase) after IN administration than after IV dosage. This may be correlated with the higher MRT values of STP obtained in the plasma and brain after IN administration relative to those obtained after IV administration (Table 4). Consequently, a slower STP elimination, particularly in the brain, was achieved after nasal dosing, being it another contribution to the obtained %B_brain IN/IV_ values.

Comparing the concentration-time profiles obtained after the IN administration of STP with the profile obtained after the oral administration of an eightfold higher dose of STP (Figure 4), the observed differences are undeniable. In fact, as demonstrated in Supplementary Table S1, the obtained plasma and brain levels of STP were statistically higher after IN administration at nearly all time points post-dosing using all tested formulations than after dose-normalized concentrations obtained after oral administration. The analysis of the most relevant dose-normalized pharmacokinetic parameters (AUC_0-t_/Dose, AUC_inf_/Dose, and C_max_/Dose, Table 4) clearly demonstrates that both C_max_ and STP total exposure in the plasma and brain are significantly higher after IN administration than after oral dosing. Actually, after STP nasal administration, C_max_ was, on average, 37.4-fold higher in plasma and 110.6-fold higher in the brain than the C_max_ values obtained after oral administration. Similarly, the attained AUC_0-t_ values after IN administration were, on average, 9.61-fold higher in plasma and 8.37-fold higher in the brain (Table 4). The absolute oral bioavailability (F) calculated in the present study was 26.6% (Table 5), a value that is in accordance with that obtained in humans [14]. Correspondingly, the relative bioavailability (F_rel_) of each IN microemulsion was 946.6% for FS6, 892.6% for FS6 + 0.25%CH, and 1053.5% for FS6 + 0.25%CH + 1%BSA (Table 5). Consequently, the brain accumulation of STP after IN dosing, compared with oral administration (%B_brain IN/Oral_—Table 5), is even more pronounced than when IV and nasal routes are compared.

When comparing the different IN microemulsions in an in vivo context, the plasmatic and brain STP C_max_ obtained after the administration of FS6 were, respectively, 1.42-fold and 1.72-fold higher than the C_max_ values obtained with FS6 + 0.25%CH (Table 4). The AUC_0-t_ e AUC_inf_ values of STP in the plasma and brain were also, respectively, 1.12-fold and 1.08-fold higher after the IN administration of FS6 than after IN administration of FS6 + 0.25%CH (Table 4). Nevertheless, Figure 4 demonstrates a second peak of STP plasmatic and brain concentrations 45 min after administration of FS6 + 0.25%CH, a finding not observed after the IN administration of FS6. This might reveal some mucoadhesive effect of chitosan, possibly leading to prolonged nasal absorption of STP that could justifies the increase of its concentrations at a longer time postdosing. Since the addition of chitosan to FS6 might lead to a longer STP release in the nasal cavity, that could also justify the slower elimination rate of STP in the brain and plasma, consequently leading to a higher t_1/2el_ when FS6 + 0.25%CH was intranasally administered (Table 4). Still, regardless of the IN administration of FS6 and FS6 + 0.25%CH, the plasma and brain concentrations of STP strongly decreased after 2 h postdosing, reaching brain concentration values below our lower limit of quantification during analysis. Therefore, a third microemulsion containing 0.25% chitosan plus 1% albumin (FS6 + 0.25%CH + 1%BSA) was further tested in another mice group using the same STP nasal dose. The aim was to increase the brain bioavailability of STP at later times post-dosing, promoting its direct transport to brain through the nasal cavity, particularly of the STP bounded to albumin. The addition of 1% albumin to FS6 + 0.25%CH resulted in a second STP brain peak concentration 8 h after its IN administration, with statistically significant differences observed in the brain STP concentrations 6-h and 8-h post FS6 + 0.25%CH + 1%BSA nasal administration compared with FS6 and FS6 + 0.25%CH IN administration (Supplementary Table S1). The addition of 1% albumin also resulted in a 1.93-fold increase in brain AUC_0-t_ compared to the administration of FS6 + 0.25%CH, and in a 1.73-fold increase in brain AUC_0-t_ when FS6 + 0.25%CH + 1%BSA is compared with FS6 nasal administration (Table 4). Nevertheless, the total plasmatic exposure of STP was not markedly different after nasal administration of the microemulsion containing 1% albumin when compared with the other two tested microemulsions. The calculated DTE and DTP values (Table 5) also showed that the addition of 1% albumin to the mucoadhesive microemulsion may potentiate brain-targeting/direct transport of STP to the brain. In fact, the obtained DTE and DTP values for FS6 and FS6 + 0.25% are, respectively, lower than 100% and 0%, but an inversion occurred when these parameters are calculated for STP loaded in FS6 + 0.25%CH + 1%BSA (DTE equals to 116.9%; DTP equals to 14.5%, Table 5). Likewise, a higher AUC_0-t_ brain/plasma ratio was obtained with this formulation, indicating a better brain distribution than the one obtained with the other formulations. This indicates that albumin might actually bind to STP molecules, having an important role in its direct transport to the brain.

## 4. Discussion

STP is a highly unstable molecule in acidic environments, which also undergoes slow and incomplete dissolution in the gastrointestinal tract. Additionally, STP also undergoes an extensive first-pass hepatic metabolism, all this limiting its oral bioavailability [12,15]. Nevertheless, oral formulations (capsules and powder for oral suspension) continue to be the only pharmaceutical forms available for the administration of this drug [16], which demands the administration of high doses to be therapeutically effective. To counterbalance this last requirement, different formulations of STP, such as micelles [18], solid dispersions [19], nanoemulsions [12], and self-nanoemulsifying systems [15], were already published in the literature. However, all these alternatives were also intended to be orally administered, with STP continuing to be a target of first-pass metabolism and to be exposed to the gastric environment. In addition, since STP is mostly prescribed to children, swallowing difficulties and the bad taste of formulations also need to be considered. Therefore, more than an alternative formulation, a different administration route can be a reliable option to increase STP bioavailability, particularly in the brain. Having in mind that the nasal cavity is the only anatomophysiological area that directly connects the brain to the external environment, with an easy access, a pH range that secures STP stability, and a large surface area that allows an extensive STP permeability [22,51], this route can be presented as a potential alternative for STP administration, possibly allowing to use lower doses than the currently administrated.

Comparing the in vivo F_rel_ (%) results obtained in the present study with the ones obtained after oral administration of the alternative STP formulations described in the literature (i.e., 218%, 206% and 444%; Supplementary Table S2), it is shown that the three intranasal microemulsions here developed lead to markedly higher values of F_rel_ (%) (~900–1000%). Unfortunately, the studies under comparison did not report brain pharmacokinetic parameters, which would be interesting in terms of comparisons with our study. Furthermore, the present study demonstrated that irrespective of the microemulsion used for the IN administration of STP, plasma and brain C_max_, as well as the respective total exposure of STP, were significantly higher after IN administration than after oral dosing. These results become even more interesting considering that the nasal administered dose was eightfold lower than the oral dose (12.5 mg/kg vs. 100 mg/kg). Therefore, with an IN administration route and a significantly lower dose, a substantial increase in STP F_rel_ (%) was accomplished, along with an increase of brain STP biodistribution without considering its plasmatic exposure (%B_brain IN/Oral_). This came to demonstrate that, if STP is not exposed to the gastrointestinal environment and if first-pass metabolism is avoided, there is no need to administer high doses as the 50 mg/kg/day currently adopted in oral administration [16]. 

The use of a microemulsion as a formulation approach is also an advantage for IN administration. Actually, they demonstrated to be thermodynamically stable and able to incorporate high quantities of lipophilic drugs, making them high drug-strength formulations [22]. Furthermore, when the droplets are homogeneously distributed (PDI < 0.1) with sizes less than 100 nm, in addition to an increased stability of the formulation itself, the delivery of drugs from the nasal cavity to the brain becomes facilitated, preventing the exposure of drugs at nontarget organs [52]. FS6 was demonstrated to fulfil all these characteristics, which may explain not only the in vitro release results but also the in vivo data obtained. The aqueous percentage of FS6 also allowed adding a mucoadhesive polymer in the formulation composition. With that, the formulation viscosity increased, possibly enabling a longer residence time in the nasal cavity without compromising STP diffusion. Bearing in mind that high viscosities can increase the residence time of formulations in the nasal cavity, but at the same time reduce the release/diffusion of drugs [44], and considering that the maximum value described in the literature for the viscosity of IN formulations is 500 mPa·s [50], it appears that the FS6 containing 0.25% chitosan was the mucoadhesive formulation that was closest to this criterion at both environmental and nasal-cavity temperatures. Thus, chitosan was chosen as a mucoadhesive polymer in a final percentage of 0.25%, since it not only allowed to maintain the ideal viscosity, droplet size, and PDI but also because this polymer is able to reversibly open the tight junctions in the nasal epithelium, thus promoting permeability [31]. Therefore, an extended STP release over time might be promoted and, with that, a higher STP bioavailability, reducing the impact of the short t_1/2el_ of STP. Still, the in vivo results showed a more sustainable release of STP after the IN administration of FS6 + 0.25%CH, demonstrated by a second concentration peak in the plasma and brain after 45 min postdosing. Even though aiming to further prolong the release of STP after IN administration, the incorporation of 1% albumin in FS6 containing 0.25% of chitosan was also tested in vivo using the same STP dose. The results demonstrated a second STP concentration brain peak 8 h postdosing. This was also accompanied by an increase of STP total exposure in the brain without increasing it in the plasma. A possible explanation can be the ability of albumin to promote the nose-to-brain direct transport of STP, as already described in the literature [36,37,38]. In fact, the obtained DTE and DTP values after nasal administration of FS6 + 0.25%CH + 1%BSA can support this direct-transport effect of albumin, contrary to those obtained for FS6 and FS6 + 0.25CH%. Therefore, the inclusion of albumin in a mucoadhesive microemulsion may allow an increase of STP in the biophase at later times post-IN dosing and decrease the peripheral systemic exposure and its associated adverse effects. Moreover, with all the nasal formulations herein tested, the STP acidic degradation in gastric acid, together with the slow and erratic STP dissolution, can be prevented. By using IN administration, the first-pass effect is also reduced, increasing the amount of STP available to bypass the BBB that, in the end, is translated into higher STP brain concentrations. Consequently, the administered dose can be significantly decreased, avoiding the waste of drug, the conversion to a nonlinear kinetic described to occur with the increase of STP doses, as well as the systemic adverse effects and drug–drug interactions associated with high doses [14,16]. In terms of clinical practice, FS6 can be used to intranasally administer STP in emergency therapies since it allows faster and higher maximum concentrations in the plasma and brain. On the other hand, FS6 + 0.25%CH + 1%BSA can be a reliable option to introduce in chronic therapy regimens once it leads to a higher STP brain exposure together with concentration peaks at short and long times postdosing. 

## 5. Conclusions

In this study, a nasal microemulsion loaded with STP was developed and pharmaceutically characterized. The inclusion of the mucoadhesive polymer chitosan as well as the addition of 1% BSA was also the target of evaluation. The three microemulsions containing STP were intranasally administered to different mice groups and further compared with IV and oral dosing. These comparative studies showed an improved STP exposure in the brain using all three microemulsions. Moreover, using an eightfold lower dose of STP, the relative bioavailability through the IN route was significantly higher than with oral administration, the only option nowadays available on the market. Thus, it became clear that the avoidance of STP gastrointestinal passage by IN administration enables the use of much lower doses to attain higher plasmatic and brain concentrations, increasing its brain bioavailability and possible therapeutic effects. The pharmacokinetic studies also demonstrated the ability of albumin to promote, to some degree, a direct nose-to-brain passage of STP molecules. Thus, the results presented in this study can be a proof of concept that IN administration can be an advantageous alternative for the administration of poorly water soluble and acid labile drugs, as is the case of STP. In addition, it was here demonstrated that the developed microemulsions present ideal characteristics to be intranasally administered and to be loaded with unstable CNS-active drugs. Moreover, it was also demonstrated that the inclusion of a mucoadhesive excipient plus a percentage of albumin in a microemulsion with a mean droplet size less than 100 nm can be a reliable option to introduce into chronic therapy regimens using drugs with short t_1/2el_ as STP. In conclusion, since the developed STP microemulsions present favourable characteristics to intranasally administer STP, those allowed STP to quickly reach higher plasmatic and brain concentrations and superior bioavailability using significantly lower doses; this could be a promising alternative to the currently available oral therapies in both emergency and chronic contexts. This set of evidence resulting from our work can support the design of future clinical trials to demonstrate the usefulness of the IN administration of STP to humans so that its delivery to the brain can be improved with lower doses and less peripheral systemic exposure, possibly resulting in an improved clinical efficacy and/or safety.

## 6. Patents

The results here presented are included in a patent application ([agency], PPP118274). PT118274 (Boletim da Propriedade Industrial nr 79/2024): Nanosistemas lipídicos para administração intranasal. Inventors: Sara Alexandra Meirinho; Gilberto Lourenço Alves; Márcio José de Abreu Marques Rodrigues. Publication date: 22/04/2024. National Application Number: PT118274. National Filling date: 04/01/2023. Applicant: University of Beira Interior. Status: Pending.

## Figures and Tables

**Table 1:** Hydrophilic formulations were tested to determine stiripentol (STP) solubility. Results of saturated STP solubility (*n* = 3) in each formulation were obtained after 48 h agitation at 25 ± 1 °C.

Formula Code	Oil		Hydrophilic Surfactant		Cosurfactant		Aqueous Phase (%)	STP Solubility ± SD (mg/mL)
	Name	%	Name	%	Name	%		
**FS1**	Imwitor 988	5	Tween 80	8	PEG 400	2	85	1.93 ± 0.12
**FS2**	Imwitor 988	5	Tween 80	25	Propylene glycol	25	45	4.51 ± 0.32
**FS3**	Imwitor 988	15	Tween 80	35	Ethanol + Propylene glycol	8.5 + 8.5	33	75.62 ± 6.63
**FS4**	Capryol 90	5.6	Kolliphor EL	31.5	Transcutol HP	32.9	30	41.74 ± 3.25
**FS5**	Capmul MCM	5	Acconon CC-6 + Tween 20	22.5 + 7.5	---	---	65	31.27 ± 3.33
**FS6**	Capmul MCM	15	Kolliphor RH 40	26.25	Transcutol HP	8.75	50	79.06 ± 4.87
**FS7**	Mygliol 812	10	Kolliphor RH 40	40	Transcutol HP	40	10	75.05 ± 4.89

PEG 400, polyethylene glycol 400; STP, stiripentol.

**Table 2:** Pharmaceutical characterization parameters of the formulations FS6, containing or not 0.5% or 0.25% of chitosan (CH) or carbopol (CB) in the final formulation. The inclusion of 1% bovine serum albumin (BSA) to FS6 plus 0.25% chitosan was also the target of evaluation. Three measurements (*n* = 3) per formulation batch were performed.

Formulation	Mean Size ± SEM (nm) ^a^	PDI ± SEM ^a^	pH	Osmolality (mOsmol/kg) ^b^	Zero Shear Viscosity at 25 °C (mPa·s)		Zero Shear Viscosity at 32 °C (mPa·s)	
					Mean ± SEM	R^2^	Mean ± SEM	R^2^
**FS6**	13.21 ± 0.09	0.066 ± 0.009	6.2	865.0 ± 16.86	124.1 ± 0.47	--	78.9 ± 0.18	--
**FS6 + 0.5%CH**	n.d.	n.d.	5.1	1034.3 ± 12.20	898.8 ± 17.43	0.9727	673.9 ± 8.41	0.9902
**FS6 + 0.25%CH**	14.15 ± 0.12	0.103 ± 0.003	5.5	951.3 ± 22.18	535.6 ± 3.86	0.9971	281.5 ± 7.82	0.9471
**FS6 + 0.5%CB**	n.d.	n.d.	6.5	610.7 ± 36.15	966.0 ± 28.50	0.9062	666.3 ± 7.35	0.9892
**FS6 + 0.25%CB**	104.86 ± 3.12	0.243 ± 0.014	6.1	503.0 ± 34.70	658.3 ± 8.15	0.9889	388.4 ± 7.08	0.9482
**FS6 + 0.25%CH** **+ 1%BSA**	15.01 ± 0.11	0.222 ± 0.007	5.6	943.2 ± 12.03	ND	ND	ND	ND

^a^ Values measured right after formulations’ preparation. ^b^ Osmolarity of FS6, FS6 + 0.5%CH, FS6 + 0.25%CH, FS6 + 0.5%CB, and FS6 + 0.25%CB was measured after a 1:2 dilution with NaCl 0.9%. BSA, bovine serum albumin; CB, carbopol; CH, chitosan; ND, not determined; PDI, polydispersity index; R^2^, coefficient of determination; and SEM, standard error of the mean.

**Table 3:** Percentual release rates of stiripentol (STP) from the formulations studied—FS6 —containing or not 0.5% or 0.25% of chitosan (CH) or carbopol (CB) in the final formulation. A STP solution in Transcutol HP was used as a positive control of release. Calculations were made fitting the results in a zero-order kinetic model. Constants rates were calculated by applying a linear regression to the plots of time vs. drug release percentage normalized with the area of the membrane used in the assay (0.64 cm^2^) and compared using an F test.

Formulation	Percentual Release (%)		Significance Matrix between Release Rates of the Different Formulations (*p*-Values)				
	R^2^	Drug Release Rate (%∙cm^−2^∙h) ± SD	FS6 + 0.5%CH	FS6 + 0.25%CH	FS6 + 0.5%CB	FS6 + 0.25%CB	STP Solution
**FS6**	0.9099	27.15 ± 3.23	0.0005	0.0024	0.0004	0.0248	0.4367 (NS)
**FS6 + 0.5%CH**	0.9930	12.56 ± 0.40	--	0.0856 (NS)	0.6229 (NS)	0.0003	<0.0001
**FS6 + 0.25%CH**	0.9655	14.63 ± 1.05	--	--	0.0497	0.0276	0.0002
**FS6 + 0.5%CB**	0.9973	12.32 ± 0.24	--	--	--	0.0002	<0.0001
**FS6 + 0.25%CB**	0.9722	18.51 ± 1.18	--	--	--	--	0.0016
**STP solution**	0.9505	32.71 ± 3.73	--	--	--	--	--

CB, carbopol; CH, chitosan; NS, not statistically significant; STP, stiripentol.

**Table 4:** Pharmacokinetic parameters of stiripentol (STP) calculated in plasma and brain homogenates supernatants following intranasal (IN) administration of FS6, FS6 plus 0.25% chitosan (FS6 + 0.25%CH), and FS6 plus 0.25% chitosan and 1% albumin (FS6 + 0.25%CH + 1%BSA) in a dose of 12.5 mg/kg, and after intravenous (IV) (12.5 mg/kg) and oral (100 mg/kg) administration to independent mice groups (*n* = 4 per time point).

Pharmacokinetic Parameters ^a^	Plasma					Brain				
	FS6	FS6 + 0.25%CH	FS6 + 0.25%CH + 1%BSA	Oral	IV	FS6	FS6 + 0.25%CH	FS6 + 0.25%CH + 1%BSA	Oral	IV
**t_max_ (min)**	10	10	10	30	5	10	10	10	30	5
**C_max_ (ng/mL)**	23,956	16,905	19,218	4282	26,602	719 ^b^	417 ^b^	468 ^b^	39 ^b^	730 ^b^
**C_max_/Dose (ng/mL)/(mg/kg)^d^**	1916.5	1352.4	1537.4	42.8	2128.2	57.5 ^b^	33.4 ^b^	37.4 ^b^	0.4 ^b^	58.4 ^b^
**AUC_0-t_ (ng.h/mL)**	14,541	13,485	16,193	12,267	5374	313	280 ^c^	540 ^c^	361 ^c^	153 ^c^
**AUC_0-t_/Dose (ng.h/mL)/(mg/kg)^d^**	1163.3	1078.8	1295.4	122.7	429.9	25.0 ^c^	22.4 ^c^	43.2 ^c^	3.6 ^c^	12.3 ^c^
**AUC_inf_ (ng.h/mL)**	14,593	13,760	16,241	12,333	5385	364 ^c^	346 ^c^	--	526 ^c^	180 ^c^
**AUC_inf_/Dose (ng.h/mL)/(mg/kg)^d^**	1167.5	1100.76	1299.25	123.33	430.80	29.10 ^c^	27.65 ^c^	--	5.26 ^c^	14.37 ^c^
**AUC_extrap_ (%)**	0.359	1.99	0.29	0.53	0.20	14.02	18.97	--	31.4	14.60
**k_el_ (h^−1^)**	0.489	0.174	0.544	0.409	0.575	1.12	0.886	--	0.316	2.389
**t_1/2el_ (h)**	1.42	3.97	1.27	1.69	1.21	0.62	0.78	--	2.19	0.29
**MRT (h)**	1.03	1.44	1.17	2.48	0.51	0.83	1.17	--	3.43	0.38

^a^ Values estimated using the mean concentration-time profiles obtained from four different mice per time point (*n* = 4). ^b^ Values expressed in ng/g. ^c^ Values expressed in ng.h/g. ^d^ Dose-normalized pharmacokinetic parameters calculated in the plasma and brain considering an oral-administration dose of 100 mg/kg and 12.5 mg/kg for the remaining routes. AUC_0-t_, area under the concentration-time curve from time zero to the time of the last sampling; AUC_extrap_, the extrapolated area under drug concentration-time curve; AUC_inf_, the area under the drug concentration-time curve to infinite; C_max_, maximum concentration; MRT, mean residence time; t_1/2el_, apparent terminal elimination half-life; t_max_, time to reach maximum peak concentration.

**Table 5:** Calculated pharmacokinetic parameters of stiripentol after the intranasal (IN) administration of FS6, FS6 plus 0.25% chitosan (FS6 + 0.25%CH), and F30 plus 0.25% chitosan and 1% albumin (FS6 + 0.25%CH + 1%BSA) in a dose of 12.5 mg/kg, and after oral (100 mg/kg) and intravenous (12.5 mg/kg) administration to independent mice groups.

	F (%)	F_rel_ (%)	AUC_0-t_ Brain/Plasma	DTE (%)	DTP (%)	%B_brain IN/IV_	%B_brain IN/Oral_
**IN FS6**	271.0	946.6	0.0215	75.3	−32.7	203.8	693.4
**IN FS6 + 0.25%CH**	255.5	892.6	0.0208	72.6	−37.5	182.6	620.9
**IN FS6 + 0.25%CH + 1%BSA**	301.6	1053.5	0.0334	116.9	14.5	352.2	1198.2
**Oral**	26.6	--	0.0285	--	--	--	--
**IV**	--	--	0.0294	--	--	--	--

AUC_0-t_, area under the concentration-time curve from time zero to the time of the last quantifiable sampling; %B_brain IN/IV_, comparative stiripentol brain bioavailability between intranasal and intravenous routes; %B_brain IN/oral_, comparative stiripentol brain bioavailability between intranasal and oral routes; DTE, drug targeting efficiency; DTP, direct-transport percentage; F, absolute bioavailability; F_rel_, relative bioavailability; IN, intranasal; IV, intravenous.

## Data Availability

Not appliable.

## Supplementary Materials

The following supporting information can be downloaded at: https://www.mdpi.com/article/10.3390/pharmaceutics15061641/s1, Table S1: Significance matrix presenting the significant differences (*p* ˂ 0.05) in mice plasma and brain between the three intranasal formulations studied and between intranasal formulations, intravenous, and oral (normalized concentrations) administration routes at the different postadministration times. Statistical analysis performed by a two-way ANOVA with Tukey’s multiple comparisons post hoc test. Table S2: Comparison between pharmacokinetic parameters obtained in plasma after the administration of alternative formulations developed for oral administration of stiripentol (STP), with the pharmacokinetic parameters obtained in plasma after administration of the developed intranasal (IN) microemulsions FS6, FS6 plus 0.25% chitosan (FS6 + 0.25%CH), and F30 plus 0.25% chitosan and 1% albumin (FS6 + 0.25%CH + 1%BSA) in a dose of 12.5 mg/kg.
