# Supplementary material for: Intranasal Microemulsion as an Innovative and Promising Alternative to the Oral Route in Improving Stiripentol Brain Targeting
Source: Pharmaceutics. 2023 Jun 1;15(6):1641. doi: 10.3390/pharmaceutics15061641 (PMC10301887; doi:10.3390/pharmaceutics15061641)
Supplement: Supplementary file 1 [file pharmaceutics-15-01641-s001.zip › Table S1.pdf]

Table S1. Significance matrix presenting the significant differences (p < 0.05) in mice plasma and brain between the three intranasal formulations studied and between intranasal formulations, intravenous and oral (normalized concentrations) administration routes at the different post-administration times. Statistical analysis performed by a two-way ANOVA with Tukey's multiple comparisons post-hoc test.

Mice matrix.	Time (min)	Significance matrix				
		**IN formulations**	**FS6+0.25%CH**	**FS6+0.25%CH+1%BSA**	**IV**	**Oral (normalized)**
**Plasma**	**5**	**FS6**	ns	ns	<0.0001	0.0024
		**FS6+0.25%CH**	--	ns	0.0029	ns
		**FS6+0.25%CH+1%BSA**	--	--	<0.0001	0.0023
	**10**	**FS6**	ns	ns	0.0221	0.0048
		**FS6+0.25%CH**	--	ns	ns	ns
		**FS6+0.25%CH+1%BSA**	--	--	ns	ns
	**15**	**FS6**	0.0292	ns	0.0008	0.0015
		**FS6+0.25%CH**	--	ns	ns	Ns
		**FS6+0.25%CH+1%BSA**	--	--	0.0255	0.0134
	**30**	**FS6**	ns	ns	0.0469	0.0343
		**FS6+0.25%CH**	--	ns	ns	0.0395
		**FS6+0.25%CH+1%BSA**	--	--	0.0283	0.0230
	**45**	**FS6**	ns	ns	ns	ns
		**FS6+0.25%CH**	--		ns	ns
		**FS6+0.25%CH+1%BSA**	--	--	ns	ns
	**60**	**FS6**	ns	ns	ns	ns
		**FS6+0.25%CH**	--	ns	0.0332	0.0292
		**FS6+0.25%CH+1%BSA**	--	--	ns	ns
	**90**	**FS6**	ns	ns	ns	ns
		**FS6+0.25%CH**	--	ns	0.0018	0.0099
		**FS6+0.25%CH+1%BSA**	--	--	0.0401	0.0373
	**120**	**FS6**	ns	ns	0.0141	0.0183
		**FS6+0.25%CH**	--	ns	ns	ns
		**FS6+0.25%CH+1%BSA**	--	--	0.0353	0.0438
	**180**	**FS6**	ns	ns	ns	ns
		**FS6+0.25%CH**	--	0.0017	0.0011	0.0005
		**FS6+0.25%CH+1%BSA**	--	--	0.0429	ns
	**240**	**FS6**	ns	ns	ns	ns
		**FS6+0.25%CH**	--	ns	0.0278	ns
		**FS6+0.25%CH+1%BSA**	--	--	ns	ns
	**360**	**FS6**	ns	0.0029	ns	ns
		**FS6+0.25%CH**	--	ns	0.0235	0.0192
		**FS6+0.25%CH+1%BSA**	ns	ns	0.0029	0.0011
	**480**	**FS6**	ns	ns	0.0199	ns
		**FS6+0.25%CH**	--	ns	ns	ns
		**FS6+0.25%CH+1%BSA**	--	--	ns	ns
	**600**	**FS6**	ns	ns	0.0304	ns
		**FS6+0.25%CH**	--	ns	ns	ns
		**FS6+0.25%CH+1%BSA**	--	--	ns	ns
	**720**	**FS6**	ns	ns	0.0305	0.0411
		**FS6+0.25%CH**	--	ns	ns	ns
		**FS6+0.25%CH+1%BSA**	--	--	ns	ns
**Brain**	**5**	**FS6**	ns	ns	0.0105	ns
		**FS6+0.25%CH**	--	ns	0.0080	0.0251
		**FS6+0.25%CH+1%BSA**	--	--	0.0074	0.0433
	**10**	**FS6**	0.0223	ns	0.0074	0.0033
		**FS6+0.25%CH**	--	ns	ns	0.0062

		FS6+0.25%CH+1%BSA	--	--	ns	ns
	15	FS6	0.0292	ns	0.0276	0.0140
		FS6+0.25%CH	--	0.0195	ns	0.0095
		FS6+0.25%CH+1%BSA	--	--	0.0394	0.0065
	30	FS6	ns	ns	ns	0.0115
		FS6+0.25%CH	--	ns	ns	0.0318
		FS6+0.25%CH+1%BSA	--	--	ns	0.0072
	45	FS6	ns	ns	ns	ns
		FS6+0.25%CH	--	ns	ns	ns
		FS6+0.25%CH+1%BSA	--	--	ns	0.0075
	60	FS6	ns	ns	0.0389	0.0087
		FS6+0.25%CH	--	ns	0.0476	0.0142
		FS6+0.25%CH+1%BSA	--	-	ns	ns
	90	FS6	ns	ns	ns	ns
		FS6+0.25%CH	--	ns	0.0299	0.0014
		FS6+0.25%CH+1%BSA	--	--	0.0243	0.0087
	120	FS6	ns	ns	ns	ns
		FS6+0.25%CH	--	ns	0.0304	0.0065
		FS6+0.25%CH+1%BSA	--	--	ns	ns
	180	FS6	--	--	--	0.0102
		FS6+0.25%CH	--	--	--	0.0102
		FS6+0.25%CH+1%BSA	--	--	--	0.0102
	240	FS6	--	ns	--	<0.0001
		FS6+0.25%CH	--	ns	--	<0.0001
		FS6+0.25%CH+1%BSA	--	--	ns	ns
	360	FS6	--	0.0062	--	--
		FS6+0.25%CH	--	0.0062	--	--
		FS6+0.25%CH+1%BSA	--	--	0.0062	0.0062
	480	FS6	--	0.0147	--	--
		FS6+0.25%CH	--	0.0147	--	--
		FS6+0.25%CH+1%BSA	--	--	0.0147	0.0147
	600	FS6	--	--	--	--
		FS6+0.25%CH	--	--	--	--
		FS6+0.25%CH+1%BSA	--	--	--	--
	720	FS6	--	--	--	--
		FS6+0.25%CH	--	--	--	--
		FS6+0.25%CH+1%BSA	--	--	--	--
