# Supplementary material for: Intranasal Microemulsion as an Innovative and Promising Alternative to the Oral Route in Improving Stiripentol Brain Targeting
Source: Pharmaceutics. 2023 Jun 1;15(6):1641. doi: 10.3390/pharmaceutics15061641 (PMC10301887; doi:10.3390/pharmaceutics15061641)
Supplement: Supplementary file 1 [file pharmaceutics-15-01641-s001.zip › Table S2.pdf]

Table S2. Comparison between pharmacokinetic parameters obtained in plasma after the administration of alternative formulations developed for oral administration of stiripentol (STP), with the pharmacokinetic parameters obtained in plasma after administration of the developed intranasal (IN) microemulsions FS6, FS6 plus 0.25% chitosan (FS6+0.25%CH) and F30 plus 0.25% chitosan and 1% albumin (FS6+0.25%CH+1%BSA) in a dose of 12.5 mg/kg.

Formulation	Administered dose	Plasma C_{max} (ng/mL)	Plasma AUC_{0-t} (ng.h/mL)	F_{rel} (%)[a]	Ref.
Oral self-nanoemulsifying system	40 mg/kg	4048.38 ± 704.54	7754.58 ± 1489.37	218.01%	[15]
Oral nanoemulsion	27 mg/kg	6160	21060	206.2%	[12]
Oral polymeric micelles	50 mg/kg	5310 ± 1010	19770 ± 3440	444%	[18]
IN FS6	12.5 mg/kg	23955.75	14541.02	946.6%	Present study
IN FS6+0.25%CH	12.5 mg/kg	16904.76	13485.48	892.6%	Present study
IN FS6+0.25%CH+0.1%BSA	12.5 mg/kg	19218.36	16193.14	1053.5%	Present study

AUC_{0-t}, area under the concentration time-curve from time zero to the time of the last quantifiable drug concentration; C_{max}, maximum peak concentration; F_{rel}, relative bioavailability; IN, intranasal.
[a] Only Lu et. al. compared the results of its study with a dilution of the commercial Diacomit® oral suspension. The remaining studies compared it with an oral suspension of STP prepared in a 0.5% solution of carboxymethylcellulose.
